# Book Review: The End of Forgetting: Growing Up With Social Media

**DOI:** 10.3389/fpsyg.2021.656238

**Published:** 2021-05-13

**Authors:** Jing Zhang, Zhipeng Li

**Affiliations:** ^1^School of Marxism, Institute of Psychological Health, Hangzhou Dianzi University, Hangzhou, China; ^2^School of Medicine, Technische Universität Dresden, Dresden, Germany

**Keywords:** digital age, social media, data, forgetting, being forgotten, moratorium

The digital age seems to be an inevitable outcome of scientific and technological progress. The development of social media is changing our lives at an incredible speed. On the plus side, young people who grew up in the digital age can use complex technological equipment skillfully to represent and express themselves more actively, yet there are costs to pay. For example, some think that they are addicted to the Internet, lose their social skills, and have poor health. The problems caused by the digital era and social media's convenience have attracted scholars' wide attention (Bauerlein, 2001). In *The End of Forgetting: Growing Up with Social Media*, Kate Eichhorn presents her considerations about social media's adverse effects. Unlike other publications that focus on the digital generation's problems from the perspective of explicit behaviors, this book mainly regards the angle of developmental psychology, pointing out how the social media era affects children's forgetting as they grow up and thus their healthy growth.

Besides “Introduction” and “Conclusion,” the book consists of five chapters. In Chapter 1 (“Documenting Childhood before and after Social Media”), with time as the mainline, Eichhorn introduces the ways and characteristics of family or personal records of growth fragments in the eras of pre-photography, photography, family film, and social media, respectively. By comparison, she indicates that children have more opportunities for self-representation in the social media era, but meanwhile, following a large number of self-representation-related data posted online, they are perhaps losing their data's ownership. Some consequent questions are worthy of our deep consideration. Can they forget when they want to? How could that influence their development?

In Chapter 2 (“Forgetting and Being Forgotten in the Age of the Data Subject”), Eichhorn discusses the value of forgetting and being forgotten during growth. It is inevitable to make mistakes and do some foolish things during growing up. Forgetting and being forgotten are indispensable properties that enable teenagers to enjoy a time of risk-free experimentation, as the phrase “psychosocial moratorium” (Erikson, 1968) means. Nevertheless, social media threatens the moratorium of teenagers while retaining valuable growth prints. The following two chapters further explore social media's impact on forgetting and growth.

Chapter 3 (“Screens, Screen Memories, and Childhood Celebrity”) introduces the concept of “screen memory.” According to Freud, screen memory allows children to edit early memory selectively, helping them choose positive memory and alleviate childhood trauma by editing terrible memory (Mahon, 2016). However, taking online celebrities and cyberbullying as examples, Eichhorn suggests rewriting and forgetting the most painful childhood memories are becoming impracticable in the social media era. With the advent of social networks and tags, it is an unprecedented challenge for a person to forget the past and reinvent himself. In Chapter 4 (“When Tagged Subjects Leave Home”), the author introduces tagging development. Mainly used for necessary tracking at first, tags quickly evolve into binding tools combined with photos. People may unconsciously tag or be tagged on social media, and tagged photos are collected and calculated to promote facial recognition technology development. Facial recognition makes it easy for individuals to be pulled back to the past even if they want to restart their lives after many years. Therefore, putting some distance between the present selves and the past is troublesome to achieve.

Because of data traces, forgetting seems to have become impossible, so can forget to be achieved by eliminating data? Chapter 5 (“In Pursuit of Digital Disappearance”) discusses the dilemma of eliminating data. Firstly, Eichhorn believes that digital space and time are not fit for young people's moratorium. Then she explains that technology companies will deprive our data of benefits because of data's economic value, which harms our ability to forget. Thirdly, she indicates that forgetting is a psychological process and being forgotten is a social process, but social media will prevent people from forgetting and being forgotten. Finally, she mentions three possible ways to make data disappear: pay for delete, deletion via data exchange, and digital abstinence.

By discussing the relationship between forgetting, freedom, and data, Eichhorn concludes that there has never been a historical period in which young people have such a strong ability to express themselves and publish information, but the price is also huge. Due to the particularity of data storage and transmission, after entering digital media, our ability to move on from childhood and adolescence has been significantly impaired, and technological and economic changes have put everyone's forgetting and being forgotten in jeopardy.

Throughout the book, combined with technological development, the author analyzes the impact of the data age on individual or group forgetting from developmental psychology and believes that the generation growing up with social media is likely to be the end of forgetting. To protect children and adolescents' growth and ensure their psychosocial moratorium, gaining the right to delete their data is crucial. Unfortunately, although Eichhorn is aware of the importance of data ownership, her suggestions regarding making the data disappear are insufficient. The first two methods (“pay for delete” and “deletion via data exchange”) can only be implemented on the premise that the data has the same attributes as other ordinary commodities. However, the data itself is replicable and easy to spread, making it impossible to exchange them straightforwardly. As for “digital abstinence,” even Eichhorn herself does not assume it a feasible solution because restricting access to new media platforms may not be suitable for children or adults. Notwithstanding, on the whole, the book is still worth reading because it leads us to think from another perspective about the harm that the development of technology and social media may bring to children and teenagers, which may shed some light on education during the social media era.

## Author Contributions

JZ and ZL wrote the manuscript, with larger contributions by JZ. ZL then provided edits and suggestions for revision. Both authors contributed to the article and approved the submitted version.

## Conflict of Interest

The authors declare that the research was conducted in the absence of any commercial or financial relationships that could be construed as a potential conflict of interest.
